# Population-based sero-epidemiological investigation of SARS-CoV-2 infection in Somalia

**DOI:** 10.1016/j.jiph.2023.04.016

**Published:** 2023-06

**Authors:** Md Shajib Hossain, Mohamed Mohamud Derrow, Sahra Isse Mohamed, Hasan Mukhtar Abukar, Mohamed G. Qayad, Sk Md Mamunur Rahman Malik, Kumlachew Fikremariam Mengistu, Ali Abdilahi Ali Obsie, Iqbal Anwar

**Affiliations:** a Ministry of Health and Human Service, Federal Government of Somalia, Somalia; b World Health Organization, Somalia; c Ministry of Health Development, Somalia; d Obstetrics and Gynaecological Society of Bangladesh, Bangladesh

**Keywords:** COVID-19, SARS-CoV-2, Seroepidemiological studies, Health facilities, Somalia

## Abstract

**Objectives:**

To explore the burden of coronavirus disease 2019 (COVID-19) in Somalia by measuring the seroprevalence of antibodies to severe acute respiratory syndrome coronavirus 2 (SARS-CoV-2) in the general population.

**Methods:**

We recruited a convenience sample of 2751 participants from among individuals attending outpatient and inpatient departments of public health facilities, or their accompanying family members. Participants were interviewed to collect sociodemographic data and provided a blood sample. We calculated seropositivity rates overall and by sex, age group, state, residence, education and marital status. We used logistic regression analysis – odds ratios and 95% confidence intervals (CI) – to investigate sociodemographic correlates of seropositivity.

**Results:**

The overall seropositivity rate was 56.4% (95% CI 54.5–58.3%), while 8.8% of participants reported being previously diagnosed with COVID-19 by July 2021. In the regression analysis, after controlling for covariates, urban residence was significantly asscoiated with seropositivity: OR = 1.74 (95% CI: 1.19–2.55).

**Conclusions:**

Our results show a high seroprevalence rate of SARS-CoV-2 in the Somali population (56.4%), and indicate that many infections have not been captured by the country’s surveillance system resulting in considerable under-reporting.

## Introduction

As coronavirus disease (COVID-19) swept through Africa, it was predicted that the pandemic would progress rapidly and overwhelm the continent’s weak health systems. Projections by the World Health Organization (WHO) Regional Office for Africa in May 2020 estimated that 29–44 million Africans could become infected in the first year of the pandemic [1].

Similarly, Somalia was expected to be overwhelmed by COVID-19. Its fragile health system was ranked 193 out of 195 in the Global Health Security Index [2] and second most fragile in the world [3]. The first case of COVID-19 was reported in Somalia on 16 March 2020 [4]. At that time, the country had no intensive care unit beds, no ventilators and no central supply of medical oxygen in the public sector for a population of more than 15 million [4].

Although Somalia still lacks reliable estimates of the true burden of COVID-19 infection and death, similar to many other African countries, it has not reported a high number of cases or deaths in spite of difficulties of planning and implementing public health measures, such as social distancing or lockdowns, and the limited capacity for testing. As of 07 December 2022, the country had reported 27 300 cases of COVID-19 including 1 352 deaths with 4.6% of all polymerase chain reaction tests being positive since the first case was reported [5].

Many reasons have been proposed for the mismatch between expectations and reality with COVID-19 in Africa, including: the young demographic; poor testing capacity; absence of systematic surveillance or testing; stigmatization of sickness preventing people attending health facilities; milder symptomology; or other population features that could affect disease severity and outcomes [6].

COVID-19 surveillance in Somalia focuses on symptomatic patients attending health-care facilities or contacting hotline numbers. As such, the full extent of the disease, including the number of mild or asymptomatic infections, is not clear. Furthermore, the role of pre-symptomatic, asymptomatic or subclinical infections of acute respiratory syndrome coronavirus 2 (SARS-CoV-2) in virus transmission is not well understood. Therefore, case reporting may underestimate the true burden of SARS-CoV-2 infection in Somalia.

Studies in sub-Saharan Africa using seroprevalence data have shown large discrepancies between reported cases and real levels of infection [7], [8]. Ratios of seroprevalence to cumulative incidence of confirmed cases varied from 18:1–954:1 by country [9]. A global systematic review found that seroprevalence estimates were on average 18.1 times higher than the corresponding cumulative incidence of COVID-19 infections [10]. These findings indicate that “confirmed” infections are a poor indicator of the spread of infection. Population-based seroprevalence surveys provide a more accurate picture of true infections because previous infections can be more accurately detected.

Although African countries contributed to 18% of 965 studies used in a meta-analysis estimating the global seroprevalence of SARS-CoV-2 [11], only 5% of the studies selected were from countries of WHO Eastern Mediterranean Region which includes Somalia. Thus, there are large gaps in our understanding of SARS-CoV-2 virus circulation in settings with such weak health systems.

To improve our understanding of the spread of SARS-CoV-2 in Somalia, we conducted a population-based seroepidemiological study using the WHO UNITY studies protocols [12], which provide a standardized methodology for seroprevalence studies to allow comparisons over time and track the evolution of immunity in the population. We aimed to explore the population burden of COVID-19 to understand the overall burden of SARS-CoV-2 infection at national and sub-national levels, including identification of high- and low-incidence areas. The specific objectives were to: (1) measure the seroprevalence of antibodies to SARS-CoV-2 in the general population, by sex and age group, to ascertain the cumulative population immunity; and (2) estimate the fraction of asymptomatic infection in the population.

## Materials and methods

### Setting

We conducted this study in Somalia’s 18 administrative regions, including the Banadir Regional Administration and Somaliland, between March and July 2021. Middle Juba was not included for security and operational reasons.

### Study design and sample size

We designed a cross-sectional population-based seroepidemiological study based on WHO protocols for population-level SARS-CoV-2 antibody testing [13]. For sample size estimation, seroprevalence was estimated at 50% ( ± 5%). Considering 95% confidence intervals (CI), a± 5% confidence limit per age group and a design effect of 1, the minimum sample size calculated was 1920. We increased the sample to allow for 384 individuals per age group: 0–4, 5–9, 10–19, 20–29, 30–39, 40–49 and ≥ 50 years. The number of participants in each age group of each region was determined by population estimates and age structures of the regions in the Somali Health and Demographic Survey 2020 [14].

A convenience sample of participants was recruited from individuals attending outpatient and inpatient departments of public health facilities, or their accompanying family members. We excluded people attending respiratory disease clinics and, only one member per family was included. With a child, her/his parent/carer was interviewed, but the blood sample was collected from the child. Recruitment continued until the predetermined sample size was achieved. Individuals who were unwilling to participate (four persons) were excluded. Written or verbal informed consent was obtained from all adults (≥18 years), and consent from parents or caregivers was obtained for participants ≤ 17 years.

This study was conducted in accordance with the World Medical Association’s Declaration of Helsinki. The study protocol was approved by the Somali Health Authority. The selected hospital authorities and their outpatient team members were informed of the study and its objectives in advance. The methods of data collection were explained in full. A robust quality assurance process was designed to ensure accurate implementation of the procedures and to minimize data collection errors. A multiorganizational team involving WHO and the United Nations Children’s Fund (UNICEF), and led by the Ministry of Health, was formed for this purpose and the sampling sites were monitored by site supervisors. The team supervised the data and sample collection activity jointly. At the end of each day, the data collection team handed over the signed consent forms and all collected samples to the district medical officer. The samples were kept in the −20 °C freezer and dried blood spot cards were kept in a dry place. When all the samples were collected, the district medical officer organized and shipped all samples and signed consent forms to the lead investigator or his/her representative for the region. In case of any identified error, the team notified the lead investigator immediately and solutions were provided on the spot. At the end of the exercise, the team was expected to submit a joint report to the lead investigator including the names of the sites supervised and photographic evidence of visits.

### Data collection and test procedures

Participants were interviewed by trained interviewers using a structured questionnaire. Responses were recorded electronically using the Open Data Kit tool. Information was collected on sociodemographic characteristics, current and past COVID-19 symptoms, COVID-19 test history and service utilization. The questionnaire, blood sample results, data collection and validation processes were digitized using the Open Data Kit. A unique identifier was assigned to each participant to link all samples, laboratory investigations and serum test reports with the interview data in the serosurvey. A trained phlebotomist collected a 5-mL venous blood sample from participants ≥ 1 year and 3-mL sample from infants< 1 year. Dry blood spot samples were prepared and the remaining whole blood was stored in individual transportation tubes labelled with the unique identifier and kept at 2–8 °C before transfer to regional hospital laboratories. At these laboratories, trained technicians separated the blood serum, prepared two serum aliquots and transferred the aliquots to three reference laboratories in Mogadishu, Hargeisa and Garowe, maintaining storage at 2–8 °C.

The reference laboratories used the WANTAI SARS-CoV-2 Ab enzyme-linked immunosorbent assay to test for the presence of SARS-CoV-2 antibodies in the first aliquot according to manufacturer’s instructions (Beijing Wantai Biological Pharmacy Enterprise Co., Ltd, China). These kits have been widely used in countries implementing the WHO Unity Studies protocols for detection of antibodies to SARS-CoV-2 infections. The kit has a combined IgM and IgG specificity of 97.5% and sensitivity of 96.7% for symptomatic cases [15], [16].

The results of this assay were reported using the Open Data Kit. The reference laboratories also made spot checks to ensure the quality of the dry blood spot samples, entered the quality status of the sample using the Open Data Kit, repacked the dry blood spot cards as per standard guidance for international shipments, and shipped the samples to the Centers for Disease Control and Prevention in Atlanta, USA, for further testing.

The second serum aliquots have been frozen and are stored at −70 °C for future use, if needed.

### Statistical analysis

Data were exported from the Open Data Kit, cleaned, coded, entered and analysed using the SPSS, version 24. Descriptive statistics such as frequencies and percentages were tabulated after the data were reviewed for completeness, consistency and accuracy. Crude unweighted seropositivity rates and 95% CI were calculated per region and within each age group (1–4 years, 5–14 years, 15–29 years, 30–49 years and ≥50 years). We used bivariate analysis and logistic regression analysis to investigate the sociodemographic correlates of seropositivity. The chi-squared test was used to assess the association of seroprevalence with sociodemographic characteristics.

## Results

### Sociodemographic characteristics

In total, 2751 respondents were enrolled in the study. Of these, 2597 (94.4%) provided a blood sample for testing and our analysis of the seroepidemiological data was based on these respondents. Data were missing for many variables in the questionnaire; participants with missing data for a variable were excluded from the analysis for that variable. Thus, the sample size varied throughout the analysis. All participants provided information about their sex, state of residence, area of residence (rural, urban, nomadic or camp for internally displaced people) and previous diagnosis of COVID-19. Only 56.0% (1540/2751) of questionnaire respondents (59.2% of those who gave a blood sample) provided information about their education or marital status (Table 1). Among all participants (those who completed the questionnaire only and those who provided a blood sample), 48.8% (1343/2751) were males. Their mean age was 22.99 years (standard deviation 17.34 years). We stratified all participants into seven age groups: 1218/2418 (50.3%) participants were younger than 20 years and 302/2418 (12.5%) were 50 years or older. Among adults, 20.6% (317/1540) were illiterate and 27.9% (430/1540) had a university level education (Table 1). As regards marital status, 65.1% (1003/1540) were married and most lived in urban areas (86.6%; 2382/2751). The highest number of participants was from Somaliland (836) participants; 30.4% of the full sample) while smallest number was from Galmudug (189 participants; 6.9% of the sample). Only 8.8% (242/2751) of participants reported having previously been diagnosed with COVID-19.

**Table 1:** Sociodemographic characteristics of the respondents, Somalia, 2021.

Variable	No. (%)
Sex (n = 2751)	
Male	1343 (48.8)
Female	1408 (51.2)
Age group, years (n = 2418)	
Mean age (SD)	22.9 (17.34)
1–4 5–9 10–19 20–29 30–39 40–49 ≥ 50	325 (13.4) 278 (11.5) 615 (25.4) 482 (19.9) 294 (12.2) 122 (5.0) 302 (12.5)
Education (n = 1540)	
Illiterate Primary Quran Secondary University	317 (20.6) 244 (15.8) 243 (15.8) 306 (19.9) 430 (27.9)
State (n = 2751)	
Banadir Galmudug Hirshabelle Jubbaland Puntland Somaliland South West	419 (15.2) 189 (6.9) 281 (10.2) 238 (8.7) 374 (13.6) 836 (30.4) 414 (15.0)
Residence (n = 2751)	
Camp for IDP Nomadic Rural Urban	78 (2.8) 56 (2.0) 235 (8.5) 2382 (86.6)
Marital status (n = 1540)	
Married Single	1003 (65.1) 537 (34.9)
Previously diagnosed with COVID-19 (n = 2751)	242 (8.8)

SD: Standard Deviation; IDP: internally displaced people; COVID-19: coronavirus disease 2019.

### Seroprevalence by sociodemographic characteristics

**Fig. 1:**
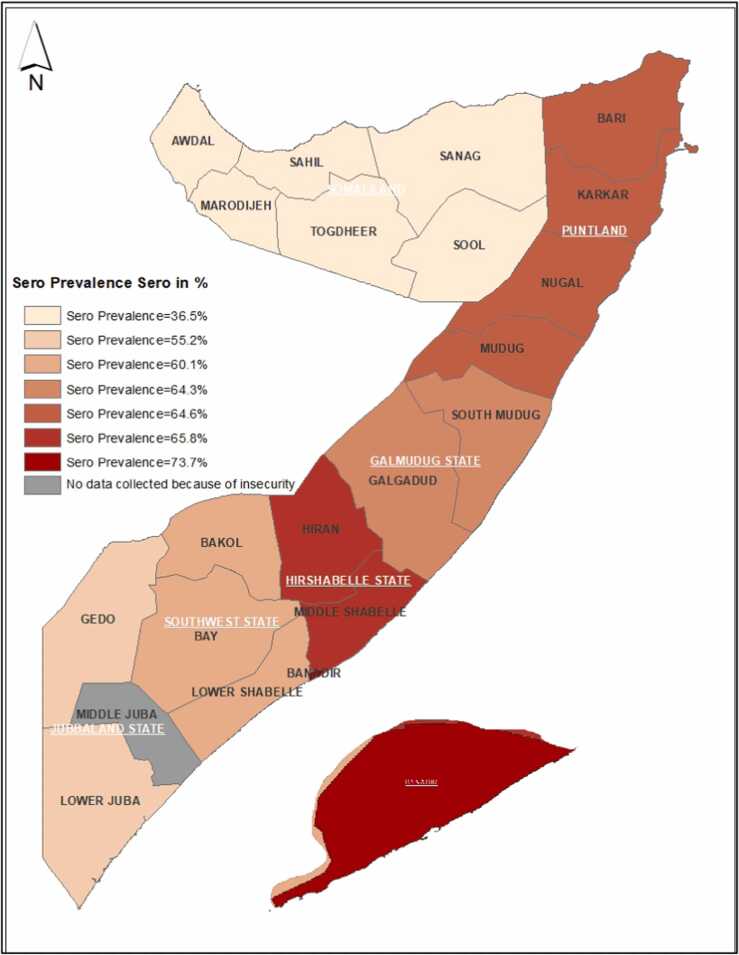
Severe acute respiratory syndrome coronavirus 2 (SARS-CoV-2) seropositivity, by state, Somalia, 2021.

The unweighted seroprevalence of SARS-CoV-2 in the study population was 56.4% (95% CI: 54.5–58.3%). In the bivariate analysis (Table 2), seropositivity was significantly higher in: males (*P* = 0.007); older age groups (*P* < 0.001); those with higher education (*P* < 0.001); and urban residents (*P* < 0.001). Seropositivity also differed significantly by state of residence (*P* < 0.001; Fig. 1) but not with marital status (*P* = 0.223). Residents of Banadir had the highest seropositivity rate (73.7%; 95% CI: 69.4–78.0%) compared with other states.

**Table 2:** Unweighted seroprevalence of SARS-CoV-2 infection, by sociodemographic characteristic, Somalia, 2021.

Characteristic	Unweighted seroprevalence, % (95% CI)	P-value
Overall (n = 2597)	56.4 (54.5–58.3)	–
Sex (n = 2597)		0.007 *
Female	53.7 (51.0–56.5)	
Male	58.9 (56.3–61.6)	
Age group, years (n = 2295)		< 0.001 *
1–4	45.8 (40.3–51.4)	
5–9	50.0 (44.0–56.0)	
10–19	58.2 (54.3–62.2)	
20–29	63.4 (58.9–67.8)	
30–39	65.5 (59.9–71.1)	
40–49	67.2 (58.7–75.8)	
≥ 50	63.0 (57.3–68.6)	
Marital status (n = 1442)		0.223
Married	61.6 (58.5–64.7)	
Single	58.3 (54.0–62.6)	
Education (n = 1442)		< 0.001 *
Illiterate	51.5 (45.8–57.2)	
Primary	61.7 (55.3–68.0)	
Quran	55.1 (48.6–61.5)	
Secondary	58.5 (52.8–64.2)	
University	71.0 (66.6–75.4)	
Residence (n = 2597)		< 0.001 *
Camp for IDP	52.0 (40.7–63.3)	
Nomadic	46.3 (33.0–59.6)	
Rural	45.0 (38.5–51.6)	
Urban	57.9 (55.8–59.9)	
State (n = 2597)		< 0.001 *
Banadir	73.7 (69.4–78.0)	
Galmudug	64.3 (57.4–71.2)	
Hirshabelle	65.8 (60.2–71.4)	
Jubbaland	55.2 (48.8–61.6)	
Puntland	64.6 (59.5–69.6)	
Somaliland	36.3 (32.9–39.8)	
South West	60.1 (55.3–64.9)	

SARS-CoV-2: severe acute respiratory syndrome coronavirus 2; IDP: internally displaced people.

* Statistically significant is *P* < 0.05.

### Factors associated with seropositivity

In the unadjusted logistic regression analysis (Table 3), males were more likely to be seropositive than females (OR=1.24; 95% CI: 1.06–1.44). No significant association was found between marital status and SARS-CoV-2 infection (Table 3). In the unadjusted analysis, SARS-CoV-2 positivity increased significantly with increasing age until age ≥ 50 years. As regards state of residence, compared with Banadir, which includes Mogadishu, residents of all other states were significantly less likely to be seropositive for SARS-CoV-2 (Table 3).

**Table 3:** Sociodemographic factors associated with SARS-CoV-2 seropositivity, Somalia, 2021: logistic regression analysis.

Independent variables	Crude OR (95% CI)	Adjusted OR (95% CI)^a^
Sex		
Female	1.00 (reference)	1.00 (reference)
Male	1.24 (1.06–1.44)	1.27 (0.97–1.66)
Age group, years		
< 0–4	1.00 (reference)	1.00 (reference)
5–9	1.18 (0.85–1.64)	2.22 (0.06–87.51)
10–19	1.65 (1.25–2.17)	0.42 (0.03–7.14)
20–29	2.04 (1.52–2.74)	0.45 (0.03–7.41)
30–39	2.24 (1.61–3.13)	0.50 (0.03–8.40)
40–49	2.43 (1.55–3.79)	0.62 (0.04–10.59)
≥ 50	2.01 (1.45–2.79)	0.52 (0.03–8.75)
Marital status		
Married	1.00 (reference)	1.00 (reference)
Single	0.87 (0.70–1.09)	0.93 (0.69–1.26)
Education		
Illiterate	1.00 (reference)	1.00 (reference)
Primary	1.52 (1.07–2.15)	1.47 (0.97–2.24)
Quran	1.15 (0.82–1.63)	0.82 (0.55–1.22)
Secondary	1.33 (0.96–1.84)	1.06 (0.71–1.58)
University	2.31 (1.69–3.15)	1.73 (1.15–2.61)*
Residence		
Rural and others^b^	1.00 (reference)	1.00 (reference)
Urban	1.65 (1.28–2.12)	1.65 (1.11–2.47)*
State		
Banadir	1.00 (reference)	1.00 (reference)
Galmudug	0.64 (0.44–0.94)	0.94 (0.51–1.75)
Hirshabelle	0.69 (0.49–0.96)	0.67 (0.41–1.09)
Jubbaland	0.44 (0.31–0.62)	0.77 (0.44–1.36)
Puntland	0.65 (0.48–0.89)	0.66 (0.41–1.04)
Somaliland	0.20 (0.16–0.27)	0.16 (0.10–0.24)
South West	0.54 (0.40–0.72)	0.46 (0.29–0.73)

SARS-CoV-2: severe acute respiratory syndrome coronavirus 2; OR: odds ratio; CI: confidence intervals.

* Statistically significant

a Adjusted for the other covariates.

b Others were people living in camps for internally displaced people and nomadic settlements.

When the effects of covariates were controlled for in the multivariate logistic regression analysis, only education and residence remained significant predictors of seropositivity (Table 3). University-educated participants were 1.73 times more likely to have tested positive for SARS-CoV-2 than those who were illiterate (OR=1.73; 95% CI: 1.15–2.61), urban residents were 1.65 (95% CI: 1.11–2.47) times more likely to be positive than rural and other residents, and residents in Somaliland and South West state were significantly less likely to have tested positive for SARS-CoV-2 than residents in Banadir (OR=0.16; 95% CI: 0.10–0.24 and OR=0.46; 95% CI: 0.29–0.73, respectively).

## Discussion

Our findings provide national seroprevalence estimates for SARS-CoV-2 infection, standardized for the general population, as described in the WHO Unity Studies protocols.

We estimated that the seroprevalence rate of SARS-CoV-2 infection in the Somali population aged ≥ 1 year was 56.4% (95% CI:54.5–58.3%). This means that by July 2021 more than half of the population had been exposed to SARS-CoV-2, an estimated 8.46 million infections since the first case of COVID-19 was reported in the country. Only 8.8% of our participants reported having been laboratory-diagnosed with COVID-19, suggesting a gross under-reporting of infections in the country.

Our finding on the seroprevalence rate of SARS-CoV-2 in Somalia concurs with the results of population-based seroprevalence studies in 19 African countries using the WHO Unity Studies protocols [9]. This study, which was a meta-analysis of 153 seroprevalence studies in Africa, found an overall seroprevalence of SARS-CoV-2 infection of 65.1% (95% CI:56.3–73.0%). The meta-analysis also found that the seroprevalence for the United Nations Eastern Africa subregion, which includes Somalia, was 63.5%, which is higher than our findings but mirrors the higher prevalence in other countries in the region. Our findings are also similar to the most recent global SARS-CoV-2 seroprevalence estimates derived from a meta-analysis of 965 studies [11].

Our study provides evidence of differences in seropositivity at both the national and state levels, and we saw statistically significant variations between different parts of the country (*P* < 0.001) ranging from 36.3% (Somaliland) to 73.7% (Banadir region). The Banadir region is the most populated administrative region and is the centre of all economic activities in the country. The first case of COVID-19 in Somalia was reported in a traveller returning to this administrative region, and more travel-associated and community-acquired cases were reported in this region than in other parts of the country. These reasons may explain the higher seroprevalence of SARS-CoV-2 in Banadir than other regions. This is in accord with a seroprevalence study conducted in Banadir region [17], which reported a seroprevalence of 44.8%, with substantial variation within the districts of this region ranging from 16% to 81%. The difference between our estimated seroprevalence rate for Banadir region and that reported by Adam et al. can be attributed to the difference in study populations, age of the respondents and the different antibody tests used.

We found that the seropositivity rate was higher in urban residents than rural populations (*P* < 0.001), and that urban residence was a significant predictor of seropositivity. Our findings are similar to those found in Sierra Leone [18], India [19] and some African countries with similar settings [9]. This difference between urban and rural settings in seroprevalence likely reflects higher contact rates in people living and sharing the same room and toilets with their extended family members in impoverished urban settings. When multiple household members live in the same house, it is challenging to maintain safe physical distancing, and such conditions have probably contributed to intense transmission of SARS-CoV-2 in such households.

Although we found a significant gender difference in SARS-CoV-2 seroprevalence (*P* < 0.007), other studies in Africa found no difference by sex [9].

The pattern of increasing seropositivity with age in our study is consistent with other studies in African countries [9], [17], [18]. We observed a higher seroprevalence rate in participants aged 20–29, 30–39 and 40–49 years but a lower rate for ≥ 50 years. This trend reflects increased intergenerational mixing at the household level and increased social interaction and economic activities of populations in these younger age groups. A similar trend was seen in the most recent global seroprevalence studies but not above 60 years [11].

While age, sex and marital status were not associated with seropositivity, university education was significantly associated. This finding is similar to that of another population-based study in India [20].

Our study confirmed that official data on the number of laboratory-confirmed cases in Somalia are a gross underestimate of the extent of community transmission in the country, a finding that mirrors those in other seroprevalence studies in Africa and worldwide [8], [9], [11]. Underestimation of the true burden of COVID-19 has been indicated by several seroprevalence studies in sub-Saharan Africa countries [6]. A plausible explanation for the discrepancy between official numbers and infections measured by our survey is the high prevalence of asymptomatic infections. The relatively young age of African populations may have resulted in more asymptomatic cases [21]. As Somalia’s testing strategies mostly focused on patients who were symptomatic and presented to health facilities, it is likely that most people with asymptomatic infections or mild infections did not visit health facilities and were hence missed in the official count of cases.

We conclude that the high seropositivity rate for SARS-CoV-2 in our study in Somalia represents a high rate of either natural or latent infection and is not attributable to vaccination. At the time the study was conducted, only 0.8% of the Somali population had received one dose of the COVID-19 vaccine and no one was fully vaccinated [22]. Therefore, the high seroprevalence we observed is an indication of the extensive spread of infection in Somalia. Despite this, many Somali people remain susceptible to SARS-CoV-2 infection because there is no consensus on antibody-based correlates of protection against SARS-CoV-2 infection. As such, the presence of antibodies is less indicative of level of protection against SARS-CoV-2 infection [23]. Furthermore, this type of immunity may induce lower anti-spike neutralizing and binding antibody responses than vaccination immunity and therefore the population may still be susceptible to infection with variants that can escape immunity, such as Omicron [24].

## Strengths and limitations

This study provides seroprevalence estimates of SARS-CoV-2 in the Somali population. Using the WHO Unity Studies protocols allows our findings to be compared with other studies using the same protocols. Our findings highlight the importance of population-based seroprevalence studies to understand the extent of community transmission of SARS-CoV-2. Somalia’s experience in conducting this study will also help improve its health system capacity to conduct such studies in the future.

Important limitations of our study were the large number of missing data in questionnaire responses for certain variables and the convenience sampling of participants. Thus, the findings cannot be generalized to the population. However, the study covered all administrative districts/regions and could give a geographical picture of COVID-19 infection. Despite our best efforts to minimize data collection errors through the quality assurance process, the complex security situation presented challenges to the procedures. For example, the supervision teams could only visit sites when local security was cleared, which limited our ability to visit all the sites during data and sample collection. Thus, quality checks of the responses provided by the data collectors could not always be done in a timely manner. The security situation also meant the survey could not be conducted at the household level using a more robust sampling methodology. Our participants were patients and their family members visiting public health facilities for non-COVID-19 reasons; therefore we cannot rule out selection bias. However, we included only one member of each family to avoid this bias. To avoid confounding, we excluded patients attending for respiratory disease symptoms. As we collected blood samples from 94.4% of the respondents and only four respondents declined to participate, we do not think these limitations led to an overestimation of the true seroprevalence. Because data were missing for some variables for participants who gave blood for testing, the differences in seropositivity by age group, residence, education and region should be interpreted with caution. However, many of our findings are comparable with other studies’ findings. Cross-reactivity with other coronaviruses may be an issue in countries in sub-Saharan Africa [25] and might lower the specificity of tests; this should be considered in the interpretation of the results. Selection bias for people more likely to be infected could occur if COVID-19 testing is not available for the general population, because people with symptoms believe they will get tested through enrolment in the study [26].

## Conclusion

Our results show a high seroprevalence rate of SARS-CoV-2 in the Somali population, possibly due to natural infection as COVID-19 vaccination coverage at the time of this study was low in the country. Although the high SARS-CoV-2 seroprevalence suggests greater population exposure and potential protection against severe COVID-19 than previously indicated by official case counts, variations were seen between geographic regions suggesting that much of the Somali population remains susceptible to SARS-CoV-2 infection. Therefore, vaccination strategies and other public health measures should target susceptible areas and age groups. Our results also indicate that many infections have not been captured by the country’s surveillance system resulting in considerable under-reporting.

## Funding

The study was funded by World Health Organization Regional Office for the Eastern Mediterranean, The 10.13039/100000865Bill and Melinda Gates Foundation, and the Foreign, Commonwealth & Development Office.

## Ethical approval

This study was conducted in accordance with the World Medical Association’s Declaration of Helsinki on ethical principles for medical research involving human subjects. The study protocol was approved by the Somali Health Authority on 11 November 2020, as there was no formal ethical review board in Somalia at the time of protocol development. The selected hospital authorities and their outpatient team members were informed of the study and its objectives in advance. The methods of data collection were explained in full – including the process of recruitment, informed consent and blood sample collection for serological investigation.

## Declaration of Competing Interest

The authors declare that they have no known competing financial interests or personal relationships that could have appeared to influence the work reported in this paper.

## References

[1] Cabore J.W., Karamagi H.C., Kipruto H., Asamani J.A., Droti B., Seydi A.B.W. (2020). The potential effects of widespread community transmission of SARS-CoV-2 infection in the World Health Organization African region: a predictive model. BMJ Glob Health.

[2] Aitken T., Chin K.L., Liew D., Ofori-Asenso R. (2020). Rethinking pandemic preparation: global health security index (GHSI) is predictive of COVID-19 burden, but in the opposite direction. J Infect.

[3] World Bank. Somalia economic update: investing in health to anchor growth [Internet]. Washington, DC: World Bank; 2021. Available from: 〈https://www.worldbank.org/en/country/somalia/publication/somalia-economic-update-investing-in-health-to-anchor-growth〉 [Accessed 3 November 2022].

[4] Ali M.M., Malik M.R., Ahmed A.Y., Bashir A.M., Mohamed A., Abdi A. (2022). Survival analysis of all critically ill patients with COVID-19 admitted to the main hospital in Mogadishu, Somalia, 30 March–12 June 2020: which interventions are proving effective in fragile states?. Int J Infect Dis.

[5] World Health Organization. Eastern Mediterranean Regional Office COVID-19 Dasboard. Cairo: World Health Organization Regional Office for the Eastern Mediterranean; 2022 [Internet] Available from: 〈https://app.powerbi.com/view?r=eyJrIjoiN2ExNWI3ZGQtZDk3My00YzE2LWFjYmQtNGMwZjk0OWQ1MjFhIiwidCI6ImY2MTBjMGI3LWJkMjQtNGIzOS04MTBiLTNkYzI4MGFmYjU5MCIsImMiOjh9〉 [Accessed 3 November 2022.

[6] Usuf E., Roca A. (2021). Seroprevalence surveys in sub-Saharan Africa: what do they tell us?. Lancet Glob Health.

[7] Kempen J.H., Abashawl A., Suga H.K., Difabachew M.N., Kempen C.J., Debele M.T. (2020). SARS-CoV-2 serosurvey in Addis Ababa, Ethiopia. Am J Trop Med Hyg.

[8] Mulenga L.B., Hines J.Z., Fwoloshi S., Chirwa L., Siwingwa M., Yingst S. (2021). Prevalence of SARS-CoV-2 in six districts in Zambia in July, 2020: a cross-sectional cluster sample survey. Lancet Glob Health.

[9] Lewis H.C., Ware H., Whelan M., Subissi L., Li Z., Ma X. (2022). SARS-CoV-2 infection in Africa: a systematic review and meta-analysis of standardised seroprevalence studies, from January 2020 to December 2021. BMJ Glob Health.

[10] Bobrovitz N., Arora R.K., Cao C., Boucher E., Liu M., Donnici C. (2021). Global seroprevalence of SARS-CoV-2 antibodies: a systematic review and meta-analysis. PLoS One.

[11] Bergeri I., Whelan M., Ware H., Subissi L., Nardone A., Lewis H.C. (2022). Global SARS-CoV-2 seroprevalence: a systematic review and meta-analysis of standardized population-based studies from Jan 2020-May. medRxiv.

[12] Bergeri I., Lewis H.C., Subissi L., Nardone A., Valenciano M., Cheng B. (2022). Early epidemiological investigations: World Health Organization UNITY protocols provide a standardized and timely international investigation framework during the COVID‐19 pandemic. Influenza Other Respir Virus.

[13] World Health Organization. The unity studies: WHO sero-epidemiological investigations protocols. Geneva: World Health Organization; 2021. Available from: https://www.who.int/emergencies/diseases/novel-coronavirus-2019/technical-guidance/early-investigations [Accessed 3 November 2022.

[14] Federal Government of Somalia. The Somali Health and Demographic Survey 2020. Mogadishu: Directorate of National Statistics, Federal Government of Somalia; 2021. Available from: https://api.nbs.gov.so/wwwroot/Surveys/6f5c03c3e73c4ff98d9b5dae1f0fab11.pdf [Accessed 3 November 2022.

[15] GeurtsvanKessel C.H., Okba N., Igloi Z., Bogers S., Embregts C.W., Laksono B.M. (2020). An evaluation of COVID-19 serological assays informs future diagnostics and exposure assessment. Nat Commun.

[16] Nyagwange J., Kutima B., Mwai K., Karanja H.K., Gitonga J.N., Mugo D. (2022). Comparative performance of WANTAI ELISA for total immunoglobulin to receptor binding protein and an ELISA for IgG to spike protein in detecting SARS-CoV-2 antibodies in Kenyan populations. J Clin Virol.

[17] Adam M.H., Mohamoud J.H., Mohamood A.S., Mohamed A.A., Garba B., Dirie N.I. (2022). Seroprevalence of anti-SARS-CoV-2 antibodies in Benadir Region, Somalia. Vaccines.

[18] Barrie M.B., Lakoh S., Kelly J.D., Kanu J.S., Squire J.S., Koroma Z. (2021). SARS-CoV-2 antibody prevalence in Sierra Leone, March 2021: a cross-sectional, nationally representative, age-stratified serosurvey. BMJ Glob Health.

[19] Murhekar M.V., Bhatnagar T., Thangaraj J.W.V., Saravanakumar V., Kumar M.S., Selvaraju S. (2021). SARS-CoV-2 seroprevalence among the general population and healthcare workers in India, December 2020–January 2021. Int J Infect Dis.

[20] Inbaraj L.R., George C.E., Chandrasingh S. (2021). Seroprevalence of COVID-19 infection in a rural district of South India: a population-based seroepidemiological study. PLoS One.

[21] Gaye B., Khoury S., Cene C.W., Kingue S., N’Guetta R., Lassale C. (2020). Socio-demographic and epidemiological consideration of Africa’s COVID-19 response: what is the possible pandemic course?. Nat Med.

[22] World Health Organization. Rolling out vaccines against COVID-19 in Somalia: scale and speed are needed. Geneva: World Health Organization; 2021. Available from: https://www.emro.who.int/images/stories/somalia/documents/covid-19-information-note-11.pdf [Accessed 2 November 2022].

[23] Earle K.A., Ambrosino D.M., Fiore-Gartland A., Goldblatt D., Gilbert P.B., Siber G.R. (2021). Evidence for antibody as a protective correlate for COVID-19 vaccines. Vaccine.

[24] Madhi S.A., Kwatra G., Myers J.E., Jassat W., Dhar N., Mukendi C.K. (2022). Population immunity and Covid-19 severity with Omicron variant in South Africa. N Engl J Med.

[25] Tso F.Y., Lidenge S.J., Peña P.B., Clegg A.A., Ngowi J.R., Mwaiselage J. (2021). High prevalence of pre-existing serological cross-reactivity against severe acute respiratory syndrome coronavirus-2 (SARS-CoV-2) in sub-Saharan Africa. Int J Infect Dis.

[26] Shakiba M., Nazemipour M., Salari A., Mehrabian F., Nazari S.S.H., Rezvani S.M. (2021). Seroprevalence of SARS-CoV-2 in guilan province, Iran, April 2020. Emerg Infect Dis.

