# Supplementary material for: Population-based sero-epidemiological investigation of SARS-CoV-2 infection in Somalia
Source: J Infect Public Health. 2023 Jun;16(6):948–54. doi: 10.1016/j.jiph.2023.04.016 (PMC10105620; doi:10.1016/j.jiph.2023.04.016)
Supplement: Supplementary file 1 — Supplementary material [file mmc1.docx]

1. **Declaration of interests**

We declare no competing interest.

1. **Funding sources**

The study was funded by World Health Organization Regional Office for the Eastern Mediterranean, The Bill and Melinda Gates Foundation, and the Foreign, Commonwealth & Development Office.

1. **Ethical approval**

This study was conducted in accordance with the World Medical Association’s Declaration of Helsinki on ethical principles for medical research involving human subjects. The study protocol was approved by the Somali Health Authority on 11 November 2020, as there was no formal ethical review board in Somalia at the time of protocol development. The selected hospital authorities and their outpatient team members were informed of the study and its objectives in advance. The methods of data collection were explained in full – including the process of recruitment, informed consent and blood sample collection for serological investigation.

1. **Acknowledgements**

This study would not have been possible without the instrumental contribution of the Minister of Health and Human service, Federal Government of Somalia, the Federal and State Ministry of Health and World Health Organization COVID-19 Incident Management support team members, the District and Regional Medical Officers of the ministry of health, the District, Regional and State Public Health Emergency Officers of World Health Organization in Somalia, the enumerators, phlebotomists and laboratory technicians collecting the data and samples in the health facilities, laboratory technicians working in Mogadishu National Public Health Reference Laboratory, Puntland State Public Health Laboratory and Somaliland Public Health Laboratory.

We also thank Ali Eman Abdelkareem, Carolina Danovaro (World Health Organization) , Nicole Hoff, Adva Gadoth (University of California, Los Angeles) for their insight and help designing the study, of the team of Laboratory Science specialists, World Health Organization (Amal Barakat, Mick Mulders, Lorenzo Subissi), of the team of data management specialists, World Health Organization (Mohamed Hussein), The Bill and Melinda Gates Foundation (Helene Martin), Biostat Global Consulting (Dhale Rhoda, Marry Kay Trimner), of the team of logistics and business operations support, World Health Organization (Hussein Hersi, Hirsi Nasir Abdi, Tom Motuku), of the team of Polio Eradication Initiative, World Health Organization (Ali Asma Swaleh, Mohammad Yusuf Elmi), The Bill and Melinda Gates Foundation (Sue Gerber, Jenna Webeck, Arie Voorman), and World Health Organization Health Emergency program (Fatima Ismail, Jama Deq Sayed).
